# *Carica papaya* induces *in vitro* thrombopoietic cytokines secretion by mesenchymal stem cells and haematopoietic cells

**DOI:** 10.1186/s12906-015-0749-6

**Published:** 2015-07-08

**Authors:** Jazli Aziz, Noor Lide Abu Kassim, Noor Hayaty Abu Kasim, Nazmul Haque, Mohammad Tariqur Rahman

**Affiliations:** Faculty of Science, International Islamic University Malaysia, Bandar Indera Mahkota, Kuantan, 25200 Malaysia; Faculty of Dentistry, International Islamic University Malaysia, Bandar Indera Mahkota, Kuantan, 25200 Malaysia; Department of Restorative Dentistry, Faculty of Dentistry, University Malaya, Kuala Lumpur, 50603 Malaysia

**Keywords:** Dengue fever, Megakaryopoiesis, Thrombocytes, Interleukin-6, Stem cell Factor

## Abstract

**Background:**

Use of Carica papaya leaf extracts, reported to improve thrombocyte counts in dengue patients, demands further analysis on the underlying mechanism of its thrombopoietic cytokines induction

**Methods:**

*In vitro* cultures of peripheral blood leukocytes (PBL) and stem cells from human exfoliated deciduous teeth (SHED) were treated with unripe papaya pulp juice (UPJ) to evaluate its potential to induce thrombopoietic cytokines (IL-6 and SCF)

**Results:**

In vitro scratch gap closure was significantly faster (*p* < .05) in SHED culture treated with UPJ. IL-6 concentration was significantly increased (*p* < .05) in SHED and PBL culture supernatant when treated with UPJ. SCF synthesis in SHED culture was also significantly increased (*p* < .05) when treated with UPJ

**Conclusion:**

*In vitro* upregulated synthesis of IL −6 and SCF both in PBL and SHED reveals the potential mechanism of unripe papaya to induce thrombopoietic cytokines synthesis in cells of hematopoietic and mesenchymal origin.

**Electronic supplementary material:**

The online version of this article (doi:10.1186/s12906-015-0749-6) contains supplementary material, which is available to authorized users.

## Background

*Carica papaya* leaves, seeds, roots and unripe pulp, have been studied for their medicinal value such as, to treat dengue fever [1, 2] and ulcer [3]; as antidiabetic and antioxidant [4], antitumor and immunomodulatory [5], wound healing [6], and antimicrobial agents [7, 8]. In relation to treat dengue, *C. papaya* leaf extract was also found to improve thrombocyte counts both in human [1] and murine animal model [2, 9]. However, the mechanism of increased thrombocyte production in response to the crude *C. papaya* leaf extract is yet to be elucidated. One possible mechanism might be attributed to the potential of papain to induce thrombocytic cytokines such as IL-6. Notably, unripe *C. papaya* is rich in proteases such as papain [10].

Purified papain was earlier reported to induce IL-6 secretion in dose dependent manner in modified mixed human lymphocyte culture [11]. Again, IL-6 stimulates thrombocyte production by increasing thrombopoietin (TPO) secretion in the liver [12]. Thus, it is expected that papaya based extracts (PBE), rich in papain [10], might also induce IL-6 and other thrombopoietic cytokines, resulting in increased thrombocyte count. Therefore, we have investigated *in vitro* thrombopoietic cytokines secretion both by human peripheral blood leukocytes (PBL) and stem cells from human exfoliated deciduous teeth (SHED) in response to unripe *C. papaya* pulp juice.

Use of the PBL and SHED has allowed to evaluate whether the potential of unripe *C. papaya* pulp juice to enhance thrombopoietic cytokines is restricted to the cells of hematopoietic origin such as PBL or, cells of other origin such as SHED can also be induced for the same. As one of the treatment strategies of thrombocytopenic diseases, secretion of megakaryopoietic or thrombopoietic affector molecules such as cytokines needs to be induced not only by the cells of hematopoietic origin but also by the cells of bone marrow, liver and kidney [12]. Therefore, the current research adds evidence that papaya have the potential to induce thrombopoietic cytokines synthesis by cells of diverse tissue origin.

## Methods

### Ethics statement

The current study required samples from healthy human donors. Therefore, this study was conducted according to the guidelines laid down in the Declaration of Helsinki and all procedures involving human subjects/patients were approved by two different Ethics committee: human deciduous dental pulp stem cells (SHED) were used with the ethical approval granted by the Faculty of Dentistry Medical Ethics Committee, University Malaya (DF CO1107/0066(L)) and peripheral blood leukocytes (PBL) were used with the ethical approval granted by the IIUM Research Ethics Committee (ID: IREC 17).Verbal consent was obtained from the PBL donors in the presence of the certified individual designated for phlebotomy and venipuncture. Written consent was obtained from the SHED donors.

### Unripe C. papaya pulp juice (UPJ) preparation

Unripe *C. papaya* was harvested from a farm in Kuantan, Malaysia. The fruit was verified by Dr. Norazian Mohd Hassan from the Kulliyyah of Pharmacy, IIUM (voucher specimen no: PIIUM 0224). The fruit was washed with distilled water, peeled, deseeded and the flesh was then blended, and the resulting pulp was squeezed through a mesh cloth to produce the unripe pulp juice (UPJ). The UPJ was centrifuged at 2000 RPM at 4°C for 15 minutes and supernatant was filtered using a sterile 0.22 μm syringe filter (Millipore, USA). Filtration sterilization was necessary to use the juice for bacterial contamination free cell culture. Other means of sterilization such as steam heat sterilization was avoided to maintain the natural composition of the juice. The sterilized UPJ was either used immediately or stored at −80°C until further use.

### Human deciduous dental pulp stem cells (SHED) culture

In brief, dental pulp was extirpated from caries free deciduous teeth (*n* = 3) of healthy patients (aged 8–11 years) undergoing extraction at the Department of Children Dentistry and Orthodontics and Department of Oral and Maxillofacial Surgery, Faculty of Dentistry University of Malaya. Following extraction, the root surfaces were cleaned with povidone-iodine (Sigma Aldrich, St Louis, MO, USA) and the teeth were then placed into sterile solution prior to sectioning. The teeth were sectioned at the cemento-enamel junction using a diamond rotary disc and the pulp were removed with an endodontic broach. The pulp were then immediately placed into sterile microcentrifuge tubes containing transportation medium and transferred to the laboratory for stem cell isolation and *in vitro* expansion as described earlier [13].

Dental pulp tissue was minced into small fragments and digested using collagenase type I (Gibco, Grand Island, NY, USA). SHEDs were cultured in Knockout™ DMEM (Gibco, USA) with 10% foetal bovine serum (Gibco, USA), 1% Glutamax (Gibco, USA), 0.5% Penicillin-Streptomycin antibiotics (Gibco, USA), and 0.5% antibiotic-antimycotic (Gibco, USA). Cells were cultured and expanded in T75 flasks under sterile conditions at 37°C and 5% CO_2_.

### Peripheral blood leukocytes (PBL) collection and culture

Human peripheral blood samples were collected in sterile tubes containing acid-citrate-dextrose (ACD), via venipuncture at the median cubital vein of healthy donors (*n* = 9). The blood components were separated by density gradient centrifugation on Lymphoprep™ (Axis-Shield, Norway). The layer of mononuclear cells was subsequently removed and washed 3 times with PBS. The resulting cell pellet was resuspended in culture media, comprising of DMEM (Nacalai Tesque, Japan), 10% foetal bovine serum (JR Scientific, USA), and 1% gentamicin-sulphate antibiotics (Nacalai Tesque, Japan). Different concentrations of UPJ i.e., 2%, 5% and 10% (v/v) were added to the media and cultured in 24-well plates. PBL cultured without UPJ addition served as the control group.

### Scratch assay

Scratch assay was performed following methods previously described [14]. SHEDs were seeded into 6-well plates and incubated at 37°C and 5% CO_2_. Once confluenced, the UPJ was added to each well according to the designated groups. A scratch was made in each well using a 200 μl pipette tip. Average gap width was measured at three different points along the scratch, using the ZEN 2011 Digital Imaging for Light Microscopy computer program (Zeiss, Germany). The surface area of the gap was computed using the ImageJ (National Institute of Health, USA). The gap width and surface area of the scratch was recorded at a 24 hour interval up to 72 hours.

### PrestoBlue® cell viability assay

Cell viability was evaluated using PrestoBlue® Cell Viability Reagent (Invitrogen, USA). SHEDs were seeded in 96-well plates and incubated 24 hours before addition of UPJ. Every 24 hours over three days, PrestoBlue® reagent was added to each well and the plates were further incubated for 2 hours at 37°C. Absorbance was measured at 570 nm with reference wavelength set at 600 nm. PrestoBlue® absorbance was used to estimate the cell viability. The absorbance values were converted to the percentage reduction of PrestoBlue® reagent using the molar extinction coefficients of the oxidized and reduced forms of the reagent. The greater the percent reduction of PrestoBlue® reagent, the higher is the cell viability.

### Total protein analysis

Concentration of total protein in PBL culture supernatant was measured using the Quick Start™ Bradford Protein Assay (Bio-Rad, USA). The culture supernatant samples and Bradford reagent were added to a 1 mL cuvette and mixed thoroughly. After allowing the mixture to settle for five minutes, absorbance was measured at 595 nm. The concentration of protein in each sample was estimated from a standard curve constructed using prepared standards from the kit.

### IL-6 and IL-3 ELISA

Concentration of IL-6 and IL-3 in PBL culture supernatant was measured using Quantikine® ELISA Kit (R&D Systems, USA). PBL culture supernatant was added to 96-well plates pre-coated with monoclonal antibodies specific for the target cytokine and incubated for two hours at room temperature. The wells were then washed followed by the addition of HRP-conjugated secondary antibodies and incubated for two hours at room temperature. The wells were again washed before adding the substrate solution. Finally, the stop solution was added after 20 minutes and absorbance was measured at 450 nm with wavelength correction set to 540 nm. Concentration of cytokines in samples was estimated from a standard curve using standards prepared from serial dilution.

### ProcartaPlex™ multiplex immunoassay

SHED culture supernatant was collected from the cultures used in the scratch assay after 24 hours and stored in −80°C until the assay was performed. A customised ProcartaPlex™ Multiplex Immunoassay (Affymetrix, USA) was used for quantitative measurements of six cytokines; interleukin-6 (IL-6), interleukin-3 (IL-3), thrombopoietin (TPO), granulocyte-macrophage colony-stimulating factor (GM-CSF), granulocyte colony-stimulating factor (G-CSF) and stem cell factor (SCF).

### Statistical analysis

Results are reported as mean ± standard error of the mean. The Wilcoxon Signed Rank test was performed to determine if there was any significant difference between the three UPJ groups and control group. The α value was set at 0.05.

## Results

### Gap closure in SHED scratch assay is enhanced by UPJ

**Fig. 1:**
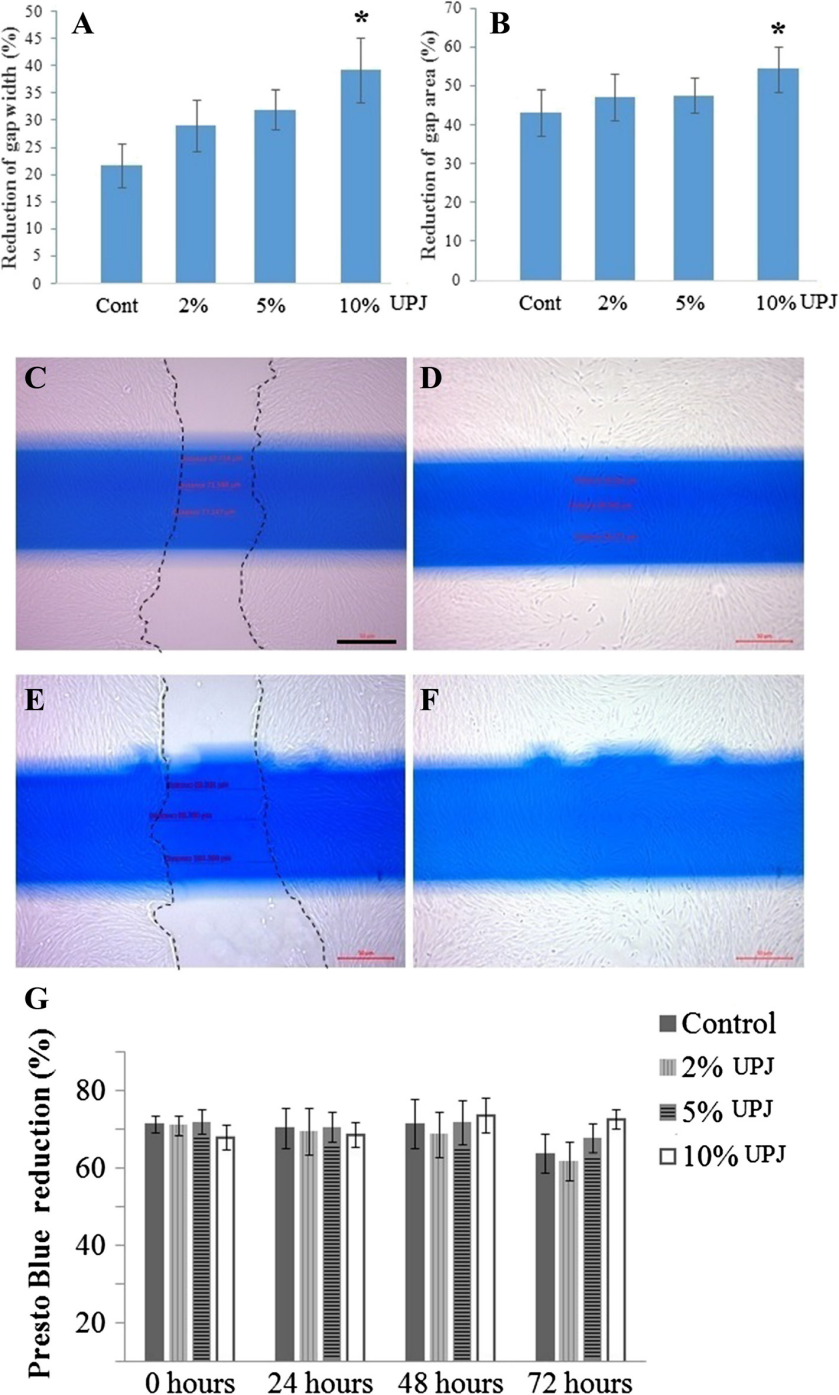
Unripe papaya pulp juice promotes gap closure *in vitro* SHED scratch culture. Addition of 10% UPJ significantly (*p* = .008) reduced scratch gap width (**a**) and gap area (**b**) compared with control groups. Addition of 2% and 5% UPJ did not show any significant difference. Representative photomicrographs of scratch assay of control and 10% UPJ groups at 0 hour (**c** and **e** respectively) and 24 hour (**d** and **f** respectively). Dotted lines (**c** and **e**) show initial scratch. *In vitro* addition of 2%, 5% and 10% UPJ did not significantly affect SHEDs’ viability (**g**). *Significantly higher than control group (*p* = .008); *n* = 9; [scale bar (C-F): 50 μm]

The width and surface area of the gap was obtained from the scratch assay to determine the effect of UPJ on SHED proliferation. Both the width (Fig. 1a) and surface area (Fig. 1b) of the gap was significantly reduced with the addition of 10% (v/v) UPJ compared to the control (*p* = .008). At 72 hours, cell viability in the 10% (v/v) UPJ group was slightly higher compared to control and other treatment groups (Fig. 1g), however the difference was not statistically significant (*p* = .173).

### Secretion of IL-6 in SHED culture is significantly enhanced by UPJ

**Fig. 2:**
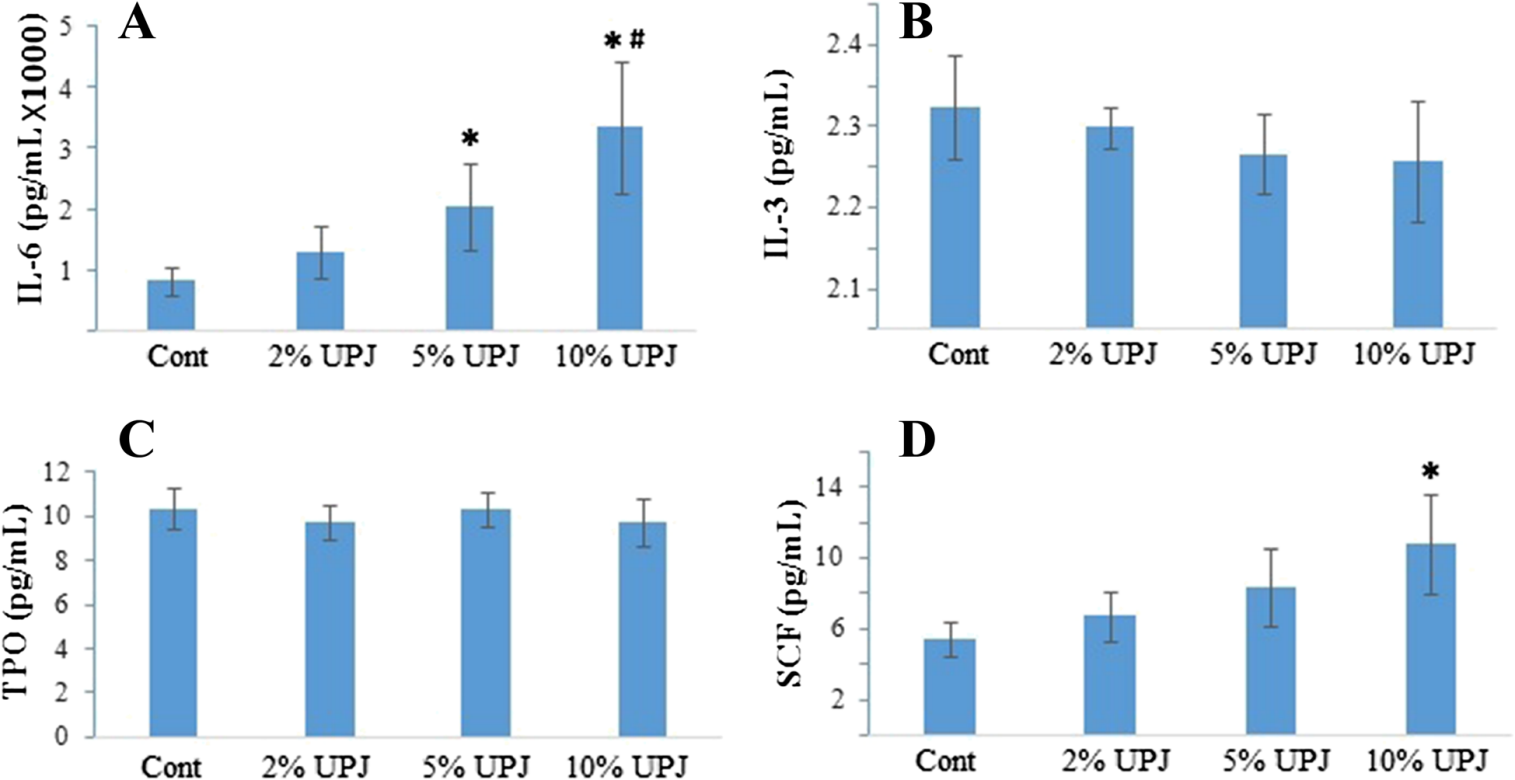
Unripe papaya pulp juice enhances in vitro IL-6 synthesis in SHED in dose dependent manner. A significant increase in IL-6 (**a**) synthesis was found in the 10% UPJ group compared to the control, 2% UPJ and 5% UPJ groups (*p* = .008), while the 5% UPJ group produced significantly higher IL-6 compared to control and the 2% UPJ group (*p* = .008). Synthesis of IL-3 (**b**), TPO (**c**) and SCF (**d**) was not significantly different between groups. *Significantly higher compared to control and 2% UPJ group (*p* = .008); ^#^Significantly higher than 5% UPJ group (*p* = .008); *n* = 9

Of the six cytokines measured, only IL-6, IL-3, TPO and SCF were detectable in the assay (Fig. 2), while G-CSF and GM-CSF were below the assay’s level of detection. IL-6 was significantly increased (*p* = .008) with the addition of 5% and 10% UPJ (Fig. 2a). increase of SCF secretion compared to the control after addition of 5% and 10% UPJ was statistically significant (*p* = .046 and *p* = .018 respectively) (Fig. 2d). Concentration of IL-3 declined with the addition of higher concentrations of UPJ (Fig. 2b) while concentration of TPO remained relatively constant across all groups (Fig. 2c).

### Enhanced IL-6 secretion in PBL culture with 2% UPJ

**Fig. 3:**
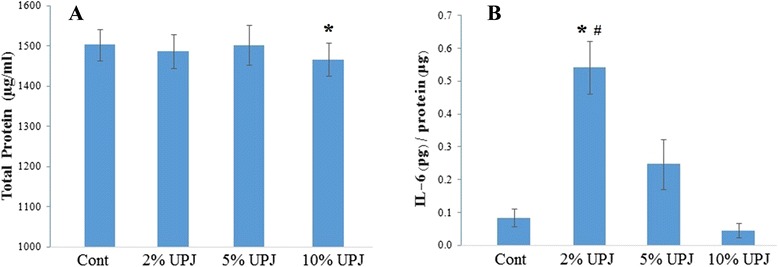
Unripe papaya pulp jucie enhances in vitro IL-6 secretion by human PBL. A significantly increased amount (*p* = .008) of IL-6 was found in supernatant of the in vitro PBL culture added with 2% UPJ compared to control and the 10% UPJ groups (**b**). Total protein content in the PBL culture supernatant added with 10% UPJ was significantly lower (*p* = .008) compared to control group (**a**). *Significantly higher compared to control group (*p* = .008); ^#^Significantly higher compared to 10% UPJ group (*p* = .008); *n* = 9

Concentration of IL-6 and IL-3 were measured in PBL culture supernatants. IL-6 concentration was significantly increased with the addition of 2% UPJ (*p* = .008). However, IL-6 concentration decreased with the addition of higher concentration of UPJ (Fig. 3b). On the other hand, total protein concentration was significantly decreased with the addition of 10% UPJ (*p* = .008) (Fig. 3a). IL-3 was not detectable by the assay.

## Discussion

Papaya leaves extract, rich in papain, was shown to improve thrombocyte (platelet) count in dengue patients [2] as well as in murine animal model [9]. Together, these observations compel further research on the underlying mechanism of thrombopoietic potential of PBE. The most likely mechanism could be linked with the upregulated thrombopoietic cytokines such as IL-6, SCF, IL-3 and TPO in response to PBEs, of which a major bioactive constituent is papain [10]. Earlier, purified papain was shown to induce IL-6 synthesis in a dose dependent manner in modified mixed lymphocyte culture [11]. In relation to the use of papaya leaf extract to treat dengue, it is important to ascertain if the PBE, similar to papain, can also induce thrombopoietic cytokines.

Therefore, the primary objective of this study is to demonstrate potential of PBE to induce thrombopoietic cytokines by the cells of the hematopoieteic origin. It is also important to evaluate whether the thrombopoietic potential of PBE is restricted to the cells of hematopoietic origin, since different thrombopoietic cytokines are synthesized by cells of different tissue origin such as IL-6 by cells of the circulatory system and TPO by the kidney [12]. Therefore, in the current study we have investigated *in vitro* induction of thrombopoietic cytokines by UPJ both in human PBLs of hematopoitic origin and in SHED, origin of which is entirely different from the hematopoietic cells [15, 16]. SHED has been an convenient source since it does not involve invasive route of collection. It is hoped that the current study would encourage furhter research using other potential organs involved in hematopoiesis such as bone marrow, kidney and liver.

Notably, SHED has been reproted to share characteristics of the mesenchymal stem cells (MSC) [15, 17, 18] such as plastic adherence, osteogenic and chondrogenic differentiation potential (Additional file1: Figure S1). Furthermore, MSCs in bone marrow play important role in regulating haematopoiesis [19, 20]. Therefore, increased secretion of thrombopoietic cytokines by SHED in response to UPJ would demonstrate the potential of papaya to induce thrombopoiesis involving hematopoietic as well as other tissues such as bonemarrow, kindney and liver [12].

Papain is one of the major cystein endopeptidases, which also include chymopapain, caricain and glycyl endopeptidase [10]. These four endopeptidases are found in latex, which can be found in differing amounts in the fruit, leaves and roots. With higher amounts of latex, and thus papain to be found in the unripe fruit, UPJ is expected to exert same therapeutic effects as papaya leaf.

We have observed that *in vitro* viability of SHED is not affected by the addition of until 10% (*v/v*) UPJ (Fig. 1g). Additionally, UPJ helped proliferation of SHED in a dose dependent manner and the proliferation was significantly higher compared to the control culture when treated with 10% (*v/v*) UPJ (Fig. 1A-1F). This increased rate of proliferation of SHED is significant since it has been shown that the number of HSCs in bone marrow is directly proportional to that of mesenchymal stem cells [19]. Thus, an increased rate of SHED proliferation should also lead to an increased rate of HSC proliferation, which could boost thrombocyte production. One possible mechanism for the increased proliferation could be related to papain in the UPJ which was earlier reported to shorten G_0_ phase of the cell cycle or recruit more dormant stem cells into the cell cycle [21].

We have also observed an increased IL-6 synthesis by the *in vitro* SHEDs when treated with increasing concentration of UPJ (Fig. 2a). And IL-6 synthesis was significantly higher in 10% (*v/v*) UPJ treated culture compared to the 2%, 5% (*v/v*) UPJ treated and control cultures. Under normal physiological conditions, IL-6 is synthesised in various tissues and cell types including macrophages, lymphocytes, fibroblasts, keratinocytes and osteoblasts. As a pleiotropic cytokine, IL-6 acts on hepatocytes in the liver, stimulating the increased expression of the otherwise constitutively produced thrombopoietin (TPO), one of the major cytokines to induce megakaryopoiesis or thrombopoiesis resulting in increased thrombocyte counts [12, 22].

Increased IL-6 expression could also lead to an increased rate of thrombocyte production by stimulating proliferation of multipotential haematopoietic progenitors [22, 23]. As a result of the increase in haematopoietic progenitor cell proliferation, mature megakaryocytes will increase in number, leading to a rise in thrombocyte counts. The observed enhanced cell proliferation (Fig. 1) could also be aided by an increased production of SCF (Fig. 2d). This notion is consistent with earlier reports that SCF acts in synergy with other cytokines, such as TPO to increase the proliferation of immature progenitor cells [24], this too helps in the thrombocyte production.

**Fig. 4:**
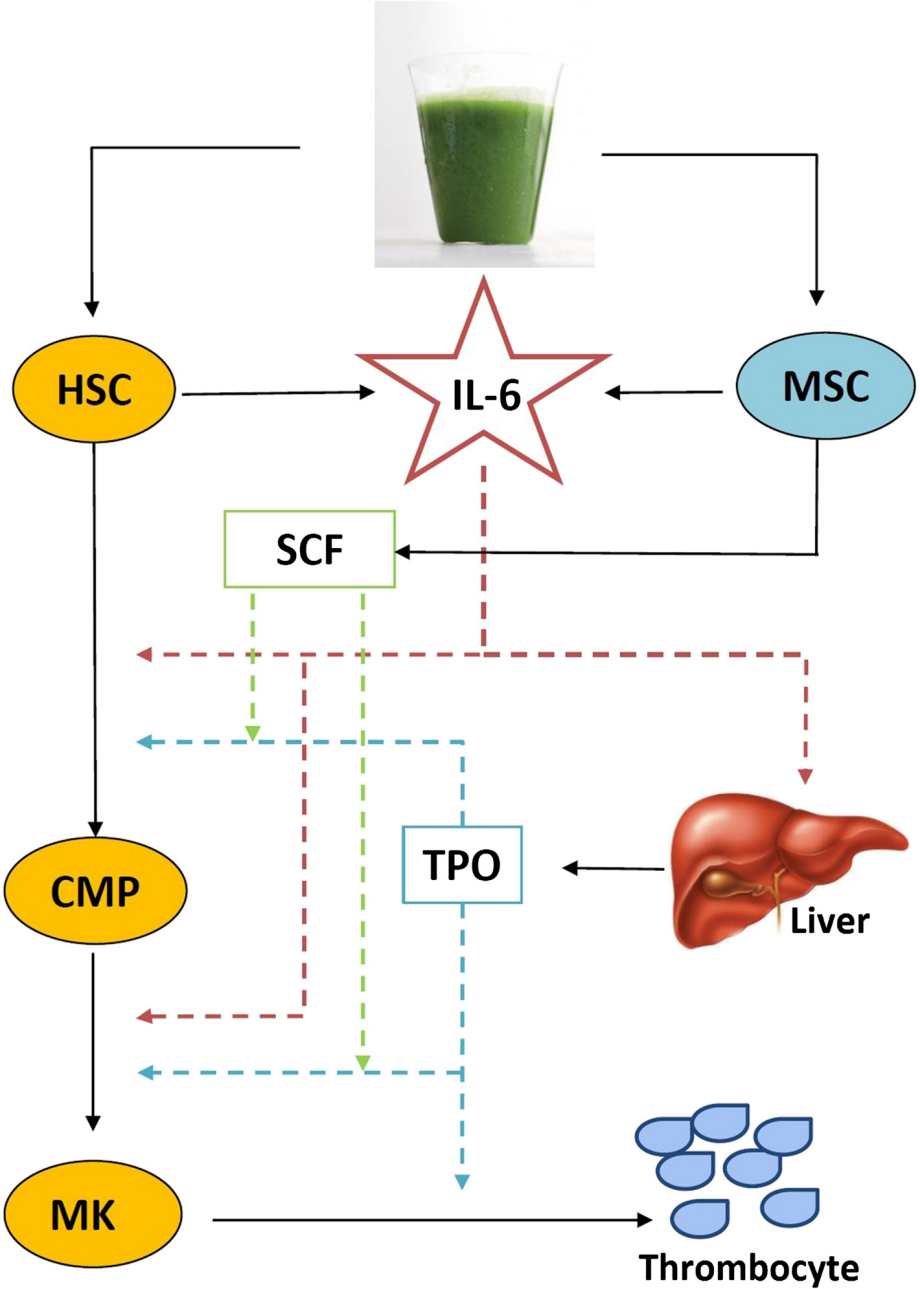
Proposed mechanism of IL-6 induced thrombocyte production. Addition of UPJ may enhance IL-6 secretion by both haematopoietic stem cells (HSC) and mesenchymal stem cells (MSC) such as SHED. IL-6 can then directly enhance HSC proliferation, maturation and differentiation, or act on the liver to stimulate higher production of TPO, the chief cytokine involved in megakaryopoiesis and thrombopoiesis. Increased synthesis of SCF by MSC may also act on TPO to further stimulate megakaryopoiesis and thrombopoiesis. [CMP: common myeloid progenitor; MK: megakaryocyte.]

Since TPO is the major cytokine involved in megakaryopoiesis and thrombopoiesis, it is possible that papaya extract/juice enhances thrombocyte production by first increasing IL-6 expression in stem cells and leukocytes, as shown in this study, which in turn enhances the production of TPO in the liver, leading to an increased rate of thrombocyte production (Fig. 4).

Unlike the dose dependent induction of IL-6 synthesis by SHEDs culture (Fig. 2a), addition of 2% (v/v) UPJ resulted in highest amount of IL-6 secretion in PBL culture supernatant compared to the addition of 10% (v/v) UPJ (Fig. 3b). Earlier it was established that induction of IL-6 synthesis may involve different pathways. Such as, IL-6 synthesis in human fibroblasts can be induced either by triggering protein kinase C [25] or by increased intracellular cyclic AMP [26]. Furthermore, induction of IL-6 synthesis can be both cell and inducer specific. For example, endotoxin is a stronger inducer in cultured monocytes than in terminally differentiated macrophages. Again, IL-1 was able to stimulate IL-6 synthesis in monocytes, but not in macrophages [27]. IL-6 synthesis was also found to be inhibited differently in different tissue, such as glucocorticosteroids prevent IL-6 gene transcription in human peripheral blood mononuclear cells [28]. In relation to PBEs, its major constituents papain and chymopapain were reported to mediate inflammation by stimulating the synthesis of prostaglandins [29]. Therefore, it is not unlikely that higher concentration of UPJ i.e., 10% (v/v) might have stronger inhibitory effect on IL-6 synthesis in PBL through stimulation of prostaglandins. At the same time it can be speculated, since similar effect is not observed, inhibition of IL-6 synthesis in SHED may follow a different pathway. Other possible clue could be the differences in number of actively proliferating cells. Compared to abundant proliferating cells in SHED culture, PBL culture is mainly composed of terminally differentiated nonproliferating lymphocytes, monocytes, macrophages and other subpopulation of leukocytes.

## Conclusion

While different medicinal plants were shown to have anti dengue virus activity [30], PBE being a strong stimulant of IL-6 and SCF, might help to improve thrombocytopenic conditions of the infected patients. Nonetheless, the use of PBE for dengue treatments needs more attention since IL-6 synthesis in monocyte culture was reported to be age dependent [31] and dengue virus sero-type specific [32]. Finally, although papain was proven to be the potent IL-6 inducer [11], it is important to link other bioactive component(s) of PBE for its thrombopoietic potential.

## Additional file

Additional file 1: Figure S1. Characterizationof the dental pulp derived SHED. Plastic adherence of SHED (A-C). Dental pulp tissue primary culture on day 7 (A); after 72 hours of incuabation, passage 3 (B); after 144 hours of incubation, passage 5 (C). Trilineage differentiation of SHED (D-F). SHEDs were cultured in chondrogenic and adipogenic differentiation medium for 14 days, and in osteogenic differentiation medium for 21 days. (D) After 14 days chondrogenic differentiation, checked by staining the cells with 'Safranin-O’. (E) After 14 days adipogenic differentiation, checked by staining with ‘Oil red O’. (F) after 21 days osteogenic differentiation, checked by staining with ‘Alizarin Red’. Photomicrographs were taken using inverted microscope (Zeiss, Germany).
