# Supplementary material for: Carica papaya induces in vitro thrombopoietic cytokines secretion by mesenchymal stem cells and haematopoietic cells
Source: BMC Complement Altern Med. 2015 Jul 8;15:215. doi: 10.1186/s12906-015-0749-6 (PMC4495848; doi:10.1186/s12906-015-0749-6)
Supplement: Additional file 1: Figure S1. — Characterizationof the dental pulp derived SHED. Plastic adherence of SHED (A-C). Dental pulp tissue primary culture on day 7 (A); after 72 hours of incuabation, passage 3 (B); after 144 hours of incubation, passage 5 (C). Trilineage differentiation of SHED (D-F). SHEDs were cultured in chondrogenic and adipogenic differentiation medium for 14 days, and in osteogenic differentiation medium for 21 days. (D) After 14 days chondrogenic differentiation, checked by staining the cells with 'Safranin-O’. (E) After 14 days adipogenic differentiation, checked by staining with ‘Oil red O’. (F) after 21 days osteogenic differentiation, checked by staining with ‘Alizarin Red’. Photomicrographs were taken using inverted microscope (Zeiss, Germany). [file 12906_2015_749_MOESM1_ESM.pdf]

Voucher specimen no.: PIIUM 0224

No.	Type of information and detail
1.	*Family name of the specimen<br>**Caricaceae**
2.	*Scientific name of the specimen, (including the authority – if any)<br>***Carica papaya* L.**
3.	*Local name<br>**Papaya/betik**
4.	*Collector's name/s (with e-mail address if any)<br>**Mr. Tan**
5.	*Student's name, matric no. & Institution<br>**Jazli Aziz, G1112139, Kulliyyah of Science, IIUM**
6.	*Date of collection<br>**April, 2013**
7.	*Plant part/s<br>**Unripe fruit**
8.	*Locality where the plant was collected, (including latitude and longitude – if any)<br>**Kuantan, Pahang**
9.	*Name of the person who determined the identification<br>**Dr. Norazian Mohd Hassan**
10.	Altitude<br>**1-600 m**
11.	*Habitat or type of plant community<br>***C. papaya* grows satisfactorily in a wide range of areas from the equatorial tropics to temperate latitudes. However, it must be grown in warm, sunny sites sheltered from wind; preferably below 150 m.**
12.	*Habit<br>**Tree-like herb**
13.	*Any other details about the plant that may be important<br>***Carica papaya* is an evergreen, tree-like herb, 2-10 m tall, usually unbranched, although sometimes branched due to injury, containing white latex in all parts. Stem cylindrical, 10-30 cm in diameter, hollow with prominent leaf scars and spongy-fibrous tissue. Has an extensive rooting system.**<br>**Leaves spirally arranged, clustered near apex of trunk; petiole up to 1 m long, hollow, greenish or purplish-green; lamina orbicular, 25-75 cm in diameter, palmate, deeply 7-lobed, glabrous, prominently veined; lobes deeply and broadly toothed.**<br>**Flowers tiny, yellow, funnel-shaped, solitary or clustered in the leaf axils, of 3 types; female flowers 3-5 cm long, large functional pistil, no stamens, ovoid-shaped ovary; male flowers on long hanging panicles, with 10 stamens in 2 rows, gynoecium absent except for a**

	**pistillode; hermaphrodite flowers larger than males, 5-carpelate ovary; occurrence depends on the season rage of the tree.**<br><br>**Fruits large (15-40 cm long, 10-30 cm diameter), cylindrical, with fleshy orange pulp, hollow berry, thin yellowish skin when ripe, varied. Fruits formed from female flowers are oblong, spherical, pear-shaped; from hermaphrodite flowers, long, obovoid or pyriform. Seeds numerous, mal, black, round, covered with gelatinous aril. Small latex vessels extend throughout the tree and are particularly abundant fruit that has reached full size but has not yet begun to ripen.**<br>**http://www.worldagroforestry.org/treedb2/AFTPDFS/Carica_papaya.pdf**
14.	*Plant/sample photos (various plant parts, preferably original photos)<br><br>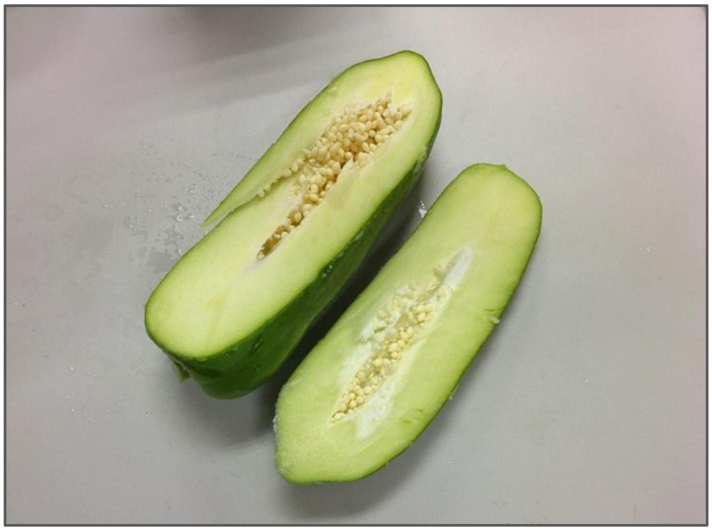<br>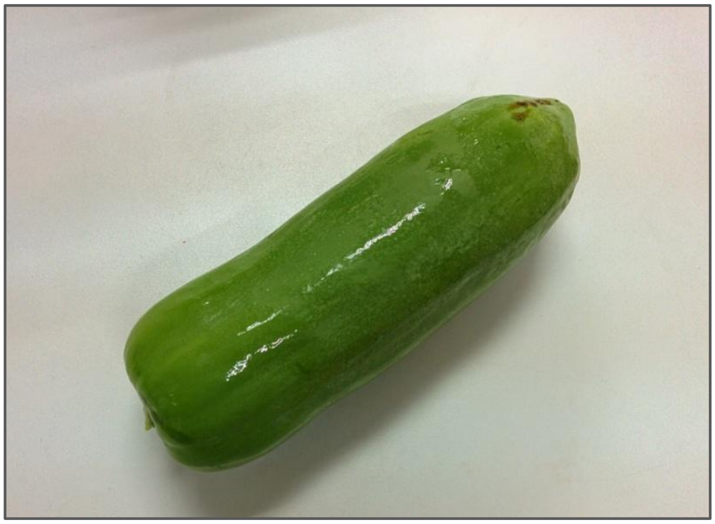<br><br>***Carica papaya* unripe fruit**
